# Supplementary material for: Dynamics of checkpoint receptors in γδ T cell subsets are associated with clinical response during anti-PD-1 immunotherapies
Source: EMBO Mol Med. 2025 Nov 27;18(1):91–119. doi: 10.1038/s44321-025-00338-9 (PMC12808275; doi:10.1038/s44321-025-00338-9)
Supplement: Supplementary file 1 — Appendix [file 44321_2025_338_MOESM1_ESM.pdf]

Dynamics of checkpoint receptors in γδ T cell subsets are associated with clinical response during anti-PD-1 immunotherapies

Elisa Catafal-Tardos[1], Lola Dachicourt[1], Maria Virginia Baglioni[1], Marcelo Gregorio Filho Fares da Silva[1], Davide Secci[1], Marco Donia[2], Anders Kverneland[2], Inge Marie Svane[2] & Vasileios Bekiaris[1]*

[1]Department of Health Technology, Technical University of Denmark, Kgs Lyngby, Denmark.

[2]National Center for Cancer Immune Therapy - CCIT-DK, Department of Oncology, Copenhagen University Hospital, Herlev, Denmark

*Corresponding author: Vasileios Bekiaris, vasbek@dtu.dk

Appendix

Appendix Figure S1. Gating strategy to identify Vδ1 and Vδ2 cells in PBMCs, control staining for ICR expression in Vδ1 and Vδ2 cells and cytokine regulation of ICR expression in Vδ1 and Vδ2 cells **2**

Appendix Figure S2. Regulation of ICR expression in human γδ T cell subsets by the JAK/STAT pathway........ **4**

Appendix Figure S3. γδ T cell responses following culture with plate-bound PDL-1 and PDL-2 or with K562 cell lines **6**

Appendix Figure S4. ICR expression in circulating γδ T cells in patients undergoing immunotherapy **7**

Appendix Figure S5. Effectiveness of integration of scRNA-seq data **9**

Appendix Figure S6. Transcriptomic analysis of circulating γδ T cell subsets from patients with melanoma and healthy donors **10**

Appendix Figure S7. γδ T cell functionality after polyclonal activation in patients with melanoma treated with monotherapy. **11**

Appendix Figure S8. γδ T cell functionality after polyclonal activation in patients with melanoma treated with combination therapy **12**

Appendix Figure S9. ICR expression in Vδ1 cells from patients with melanoma in response to TCR and IL-15 stimulation **13**

Appendix Figure S10. ICR expression in Vδ2 cells from patients with melanoma in response to TCR and IL-15 stimulation **14**

Appendix Table S1. Patient cohort in this study **15**

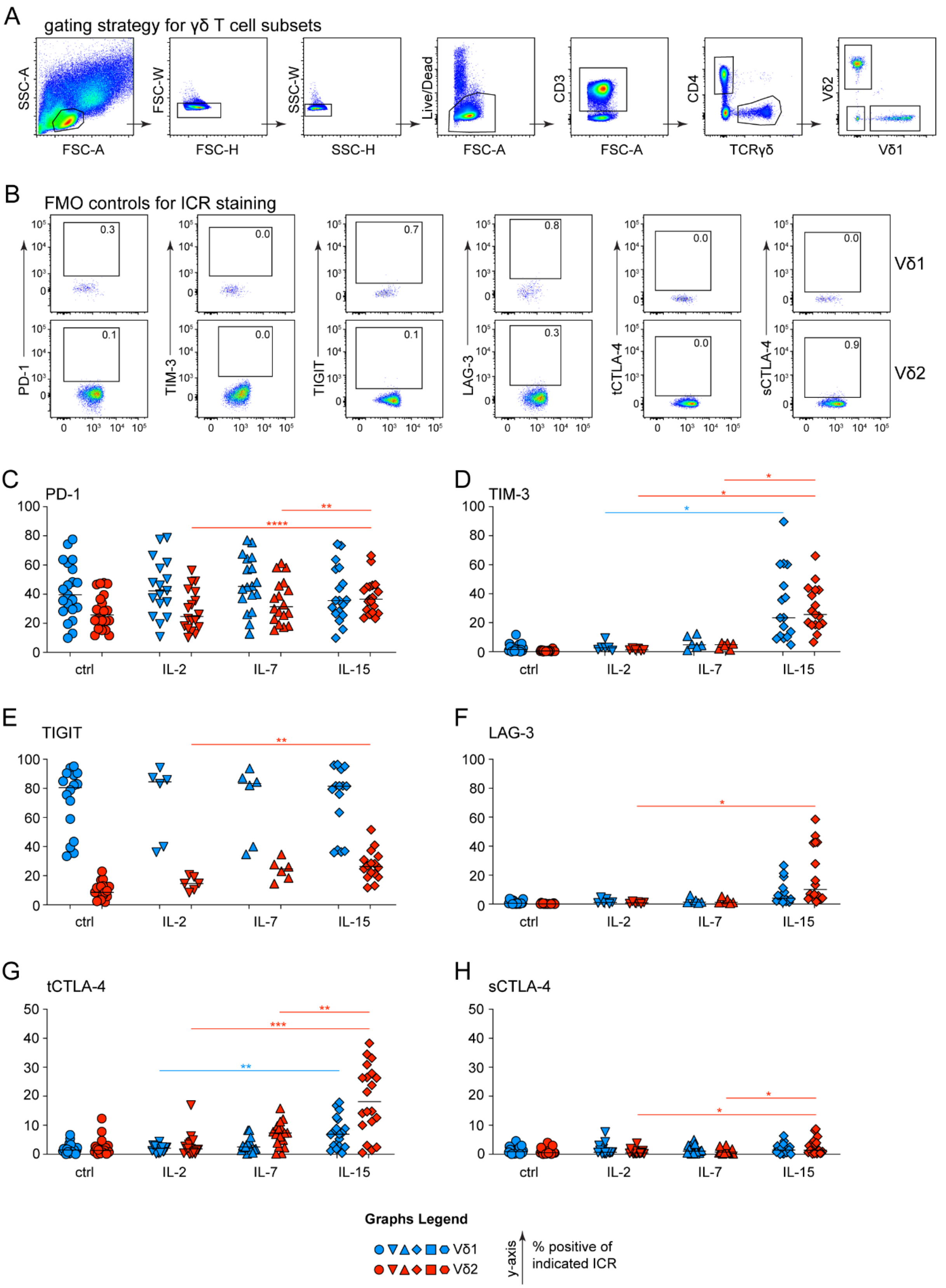

Appendix Figure S1. Gating strategy to identify Vδ1 and Vδ2 cells in PBMCs, control staining for ICR expression in Vδ1 and Vδ2 cells and cytokine regulation of ICR expression in Vδ1 and Vδ2 cells. (**A**) Gating strategy used in flow cytometry experiments to identify Vδ1 and Vδ2 cells in PBMCs. (**B**) FMO (fluorescence minus one) controls for each of the indicated α-ICR antibodies used

to measure expression of the respective ICR molecules in Vδ1 and Vδ2 cells. (**C-H**) PBMCs from 22 healthy donors were stimulated for 48h with IL-2, IL-7 or IL-15 or left untreated (ctrl). Expression of PD-1 (**C**), TIM-3 (**D**), TIGIT (**E**), LAG-3 (**F**), total (t) CTLA-4 (**G**) and surface (s) CTLA-4 (**H**) in Vδ1 (blue) and Vδ2 (red) cells. *P < 0.05, **P < 0.01, ***P < 0.001 ****P < 0.0001 by mixed-effect model with the Geisser-Greenhouse correction and Tukey's multiple comparisons test. In graphs, each symbol represents a donor.

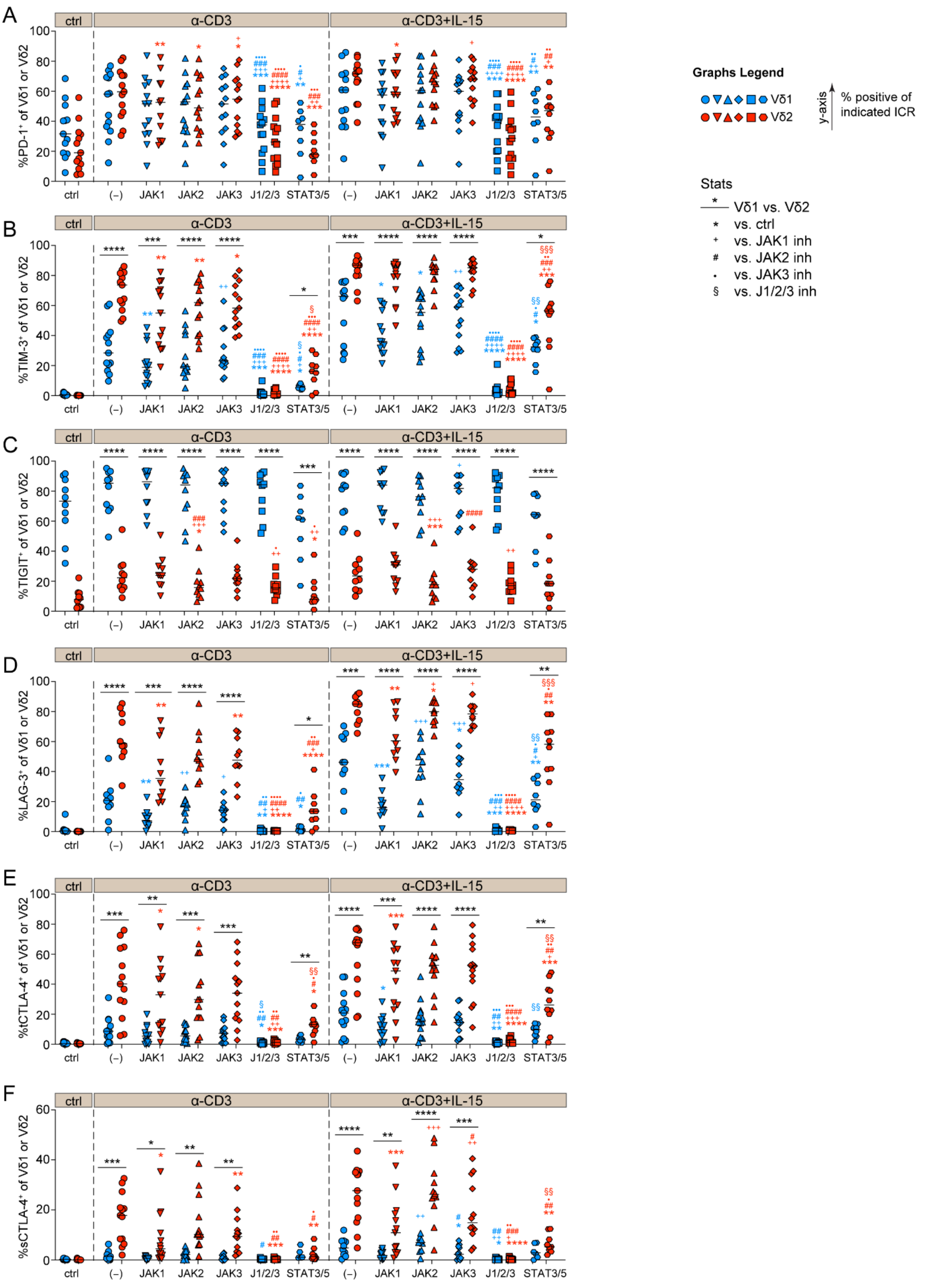

Appendix Figure S2. Regulation of ICR expression in human γδ T cell subsets by the JAK/STAT pathway. Flow cytometry analysis of PD-1 (**A**), TIM-3 (**B**), TIGIT (**C**), LAG-3 (**D**), total (t) CTLA-4 (**E**) and surface (s) CTLA-4 (**F**) in Vδ1 and Vδ2 cells. PMBCs were stimulated for 48h with α-CD3 or α-CD3 and IL-15 in the presence of inhibitors as follows. Abrocitinib was used for JAK1

inhibition; NSC33994 for JAK2 inhibition; JPX-0700 for STAT3/5 dual inhibition; and tofacitinib citrate was used at a low concertation 10 nM to inhibit JAK3 signaling, and at a high concentration of 1 μM for combined JAK1/2/3 inhibition. *P < 0.05, **P < 0.01, ***P < 0.001 ****P < 0.0001 by mixed-effect model with the Geisser-Greenhouse correction and Tukey's multiple comparisons test. $\underline{*}$ P between Vδ1 and Vδ2. * P versus control, $^{+}$ P versus JAK1 inhibition, # P versus JAK2 inhibition, ● P versus JAK3 inhibition, § P versus JAK1/2/3 inhibition in Vδ1 (blue) or red in Vδ2 (red). Each symbol represents a donor.

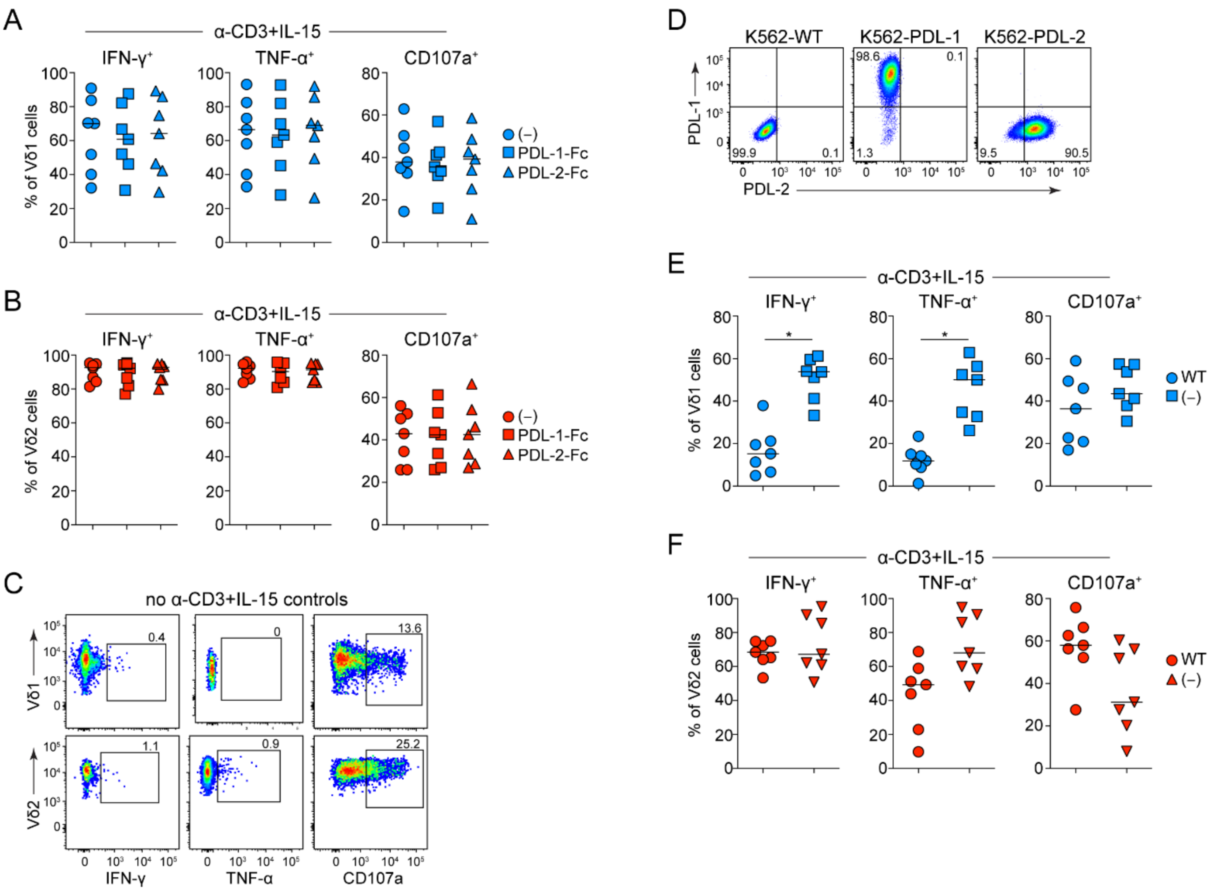

Appendix Figure S3. γδ T cell responses following culture with plate-bound PDL-1 and PDL-2 or with K562 cell lines. (**A-C**) PBMCs were magnetically depleted of $CD4^+$, $CD19^+$ and $CD14^+$ cells and cultured overnight in the absence (□) or presence of plate-bound recombinant PDL-1-Fc or PDL-2-Fc proteins while being stimulated with α-CD3 and IL-15. Flow cytometric analysis of IFN-γ, TNF-α and CD107a in Vδ1 (**A**) and Vδ2 (**B**) cells. (**C**) Representative flow cytometry plots indicating expression of IFN-γ, TNF-α and CD107a in the absence of α-CD3 and IL-15 stimulation or PDL-Fc proteins. (**D**) Representative flow cytometry plots indicating expression of PDL-1 and PDL-2 in the K562 cell lines used in main **Fig. 3**; WT = wild-type. (**E-F**) PBMCs were magnetically depleted of $CD4^+$, $CD19^+$ and $CD14^+$ cells and cultured for 48h without (□) or with WT K562 cells in the presence of IL-15 and α-CD3 stimulation. Levels of IFN-γ, TNF-α and CD107a were assessed by flow cytometry in Vδ1 (**E**) and Vδ2 (**F**) cells. *P < 0.05 by paired Wilcoxon test. In (**A-B**) statistical analysis by paired Friedman test with Dunn's multiple comparisons test did not return statistical significance. In graphs, each symbol represents a donor.

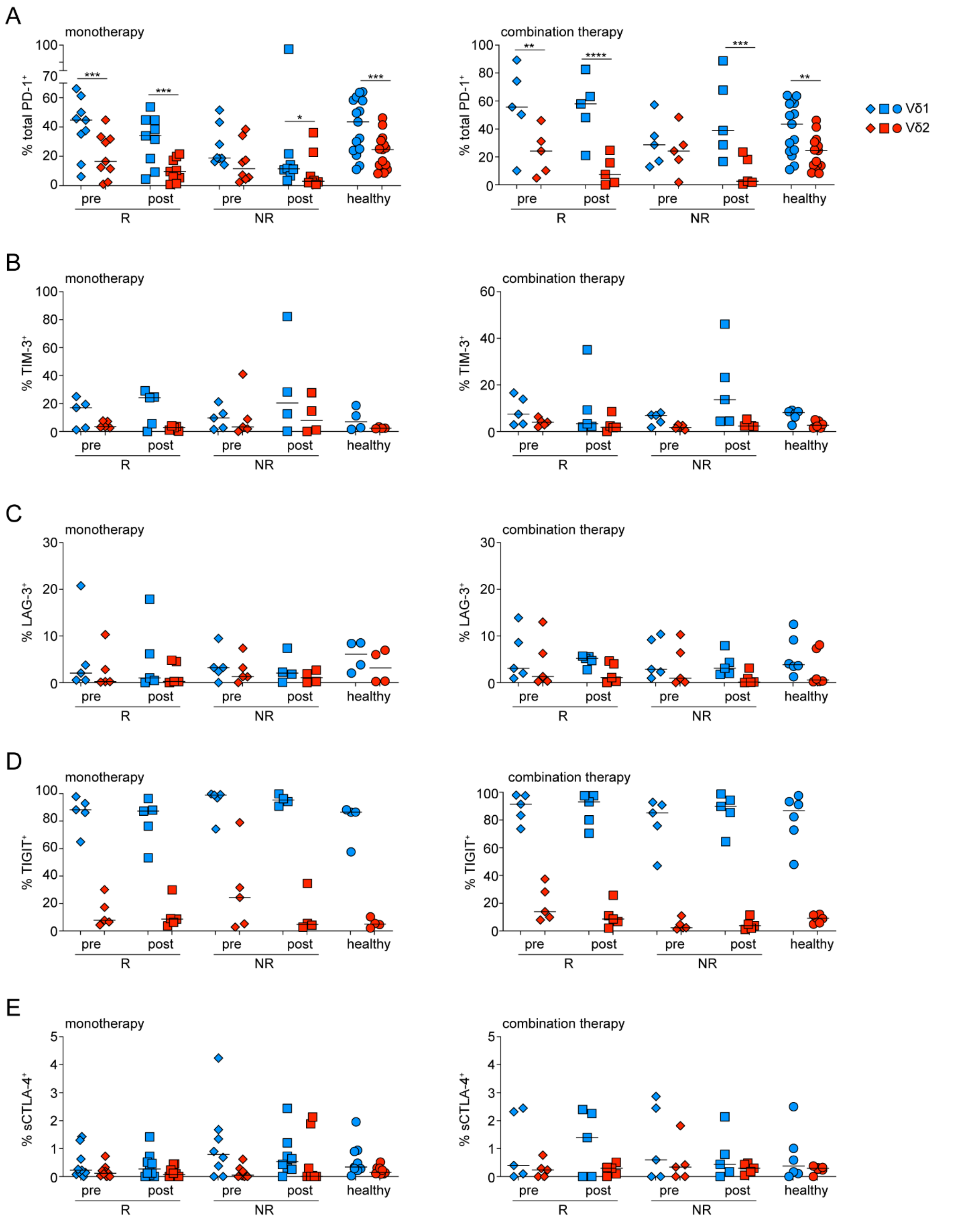

Appendix Figure S4. ICR expression in circulating γδ T cells in patients undergoing immunotherapy. Flow cytometry analysis of Vδ1 (blue) and Vδ2 (red) cells derived from patients with stage IV melanoma who were treated with either α-PD1 alone (monotherapy, left) or α-PD-1 and α-CTLA-4 (combination therapy; right). Samples were obtained from patients who responded (R) or did not respond (NR) to treatment both before (pre) and 3 to 4 months after (post) the start of immunotherapy. (**A-E**) Expression of PD-1 (**A**), TIM-3 (**B**), LAG-3 (**C**), TIGIT (**D**) and surface

(s)CTLA-4 (**E**). PBMCs from healthy donors (healthy) were used as control. *P < 0.05, **P < 0.01, ***P < 0.001 ****P < 0.0001 by paired two-way ANOVA and Tukey's multiple comparisons test. In graphs, each symbol represents a donor.

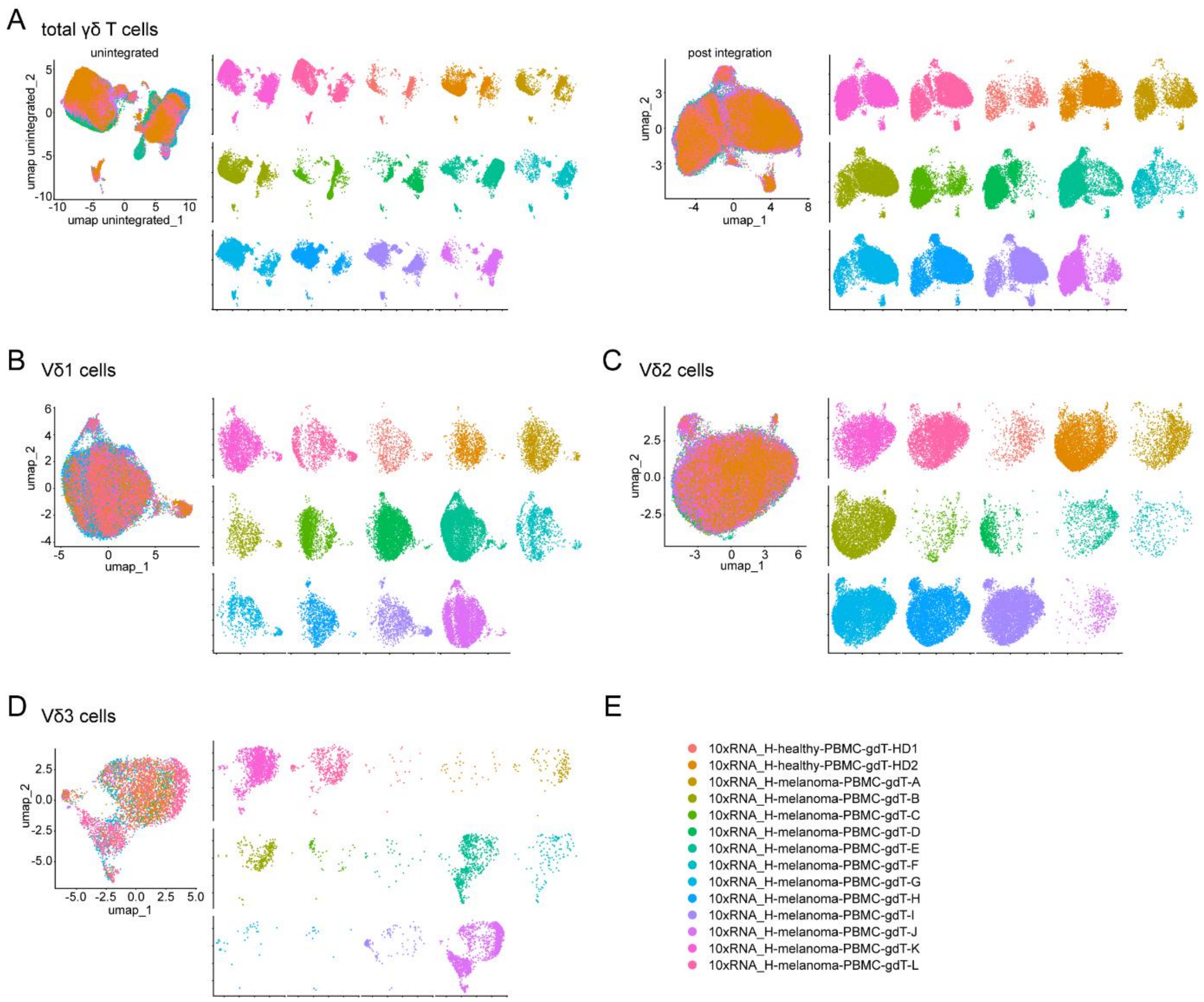

Appendix Figure S5. Effectiveness of integration of scRNA-seq data. (**A**) UMAP embedding showing clustering of circulating total γδT cells before (left) and after (right) integration. Each color represents an individual sample run on the 10x platform. (**B**-**D**) UMAP embedding showing clustering of circulating Vδ1 (**B**), Vδ2 (**C**), and Vδ3 (**D**) cells after integration. Each color indicates an individual sample run on the 10x platform. (**E**) Legend indicating sample names.

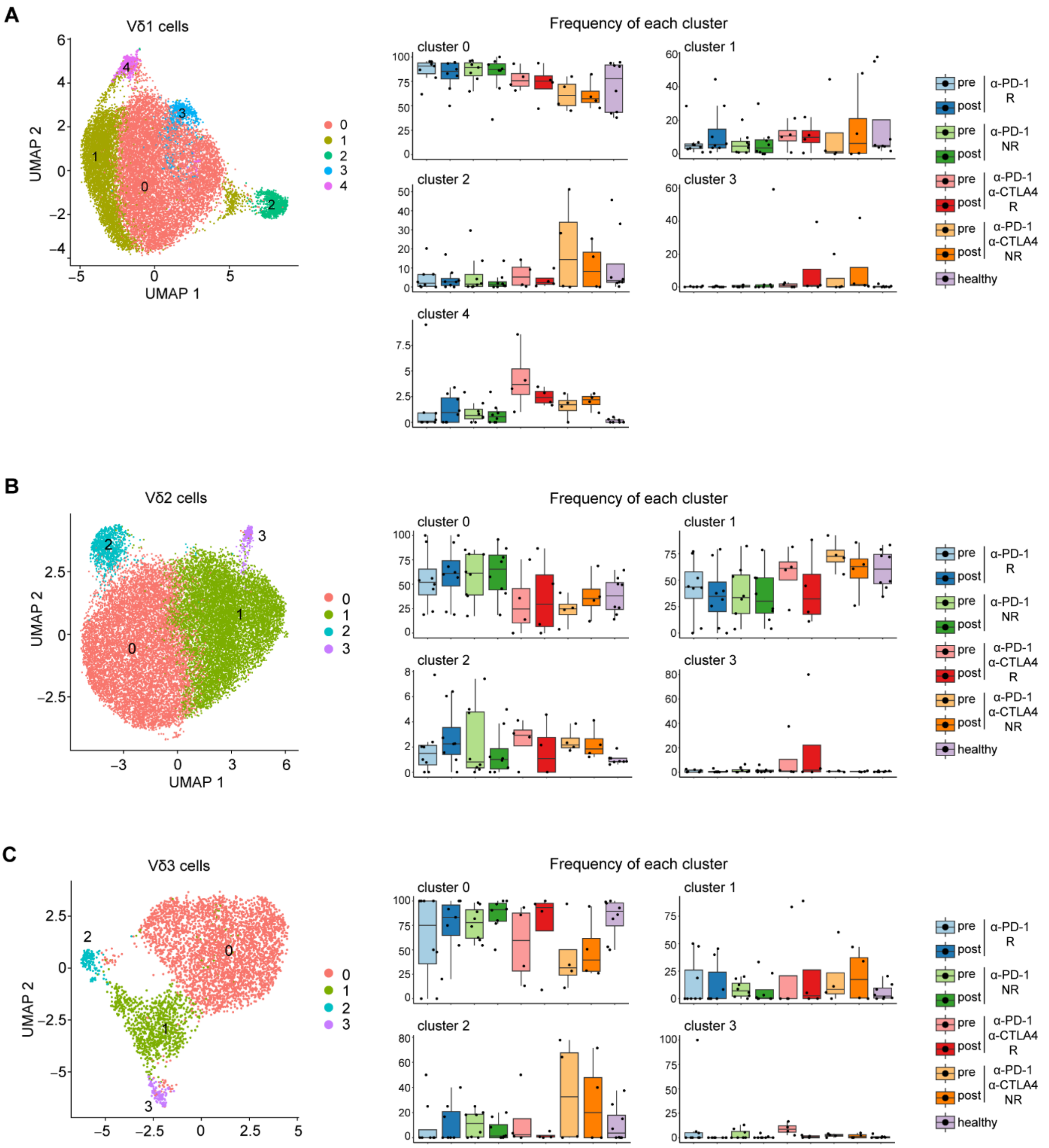

Appendix Figure S6. Transcriptomic analysis of circulating γδ T cell subsets from patients with melanoma and healthy donors. UMAP embeddings showing clustering of circulating (**A**) Vδ1 T cells (n = 27827), (**B**) Vδ2 T cells (n = 42814), and (**C**) Vδ3 T cells (n = 4937) derived from healthy donors (n = 8) and patients with stage IV melanoma who received α-PD-1 (n = 16) or a combination of α-PD-1 and α-CTLA-4 (n = 8) treatment, and the distribution of cells between clusters in each sample. To calculate the frequency of cells assigned to each cluster we only considered samples with at least 50 cells; each dot represents an individual sample. *$P < 0.05$ by paired Kruskal-Wallis test and Dunn’s correction test.

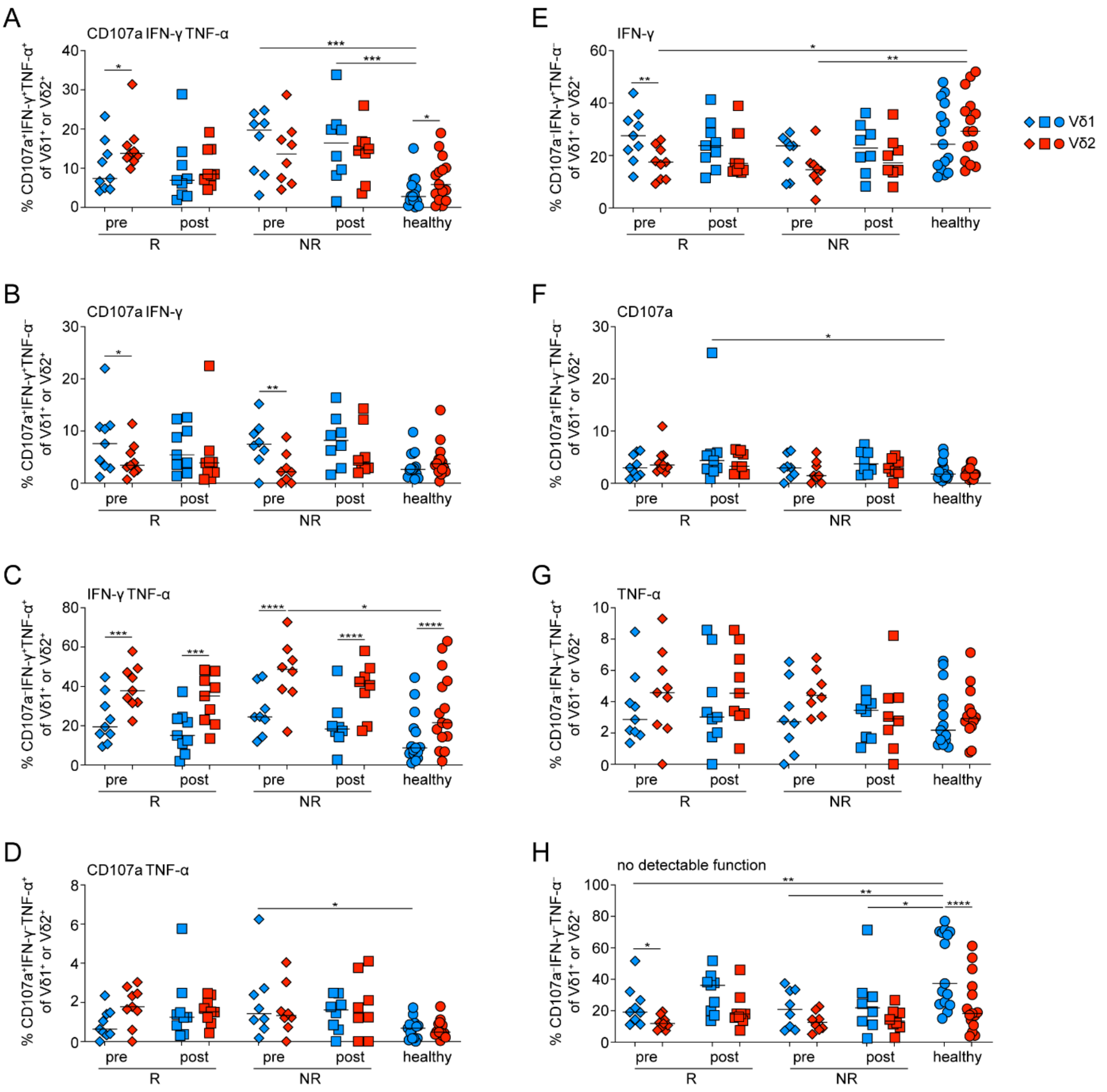

Appendix Figure S7. γδ T cell functionality after polyclonal activation in patients with melanoma treated with monotherapy. PBMCs from healthy donors (healthy) and patients with stage IV melanoma who responded (R) or did not respond (NR) to α-PD-1 treatment were stimulated for 3.5h with PMA+ionomycin to assess cytokine production. Patient samples from before (pre) and 3 to 4 months after (post) the start of treatment were analyzed. (**A**) Frequency of Vδ1 and Vδ2 cells that co-express CD107a, IFN-γ and TNF-α. (**B**) Frequency of cells that co-express CD107a and IFN-γ. (**C**) Frequency of cells that co-express IFN-γ and TNF-α. (**D**) Frequency of cells that co-express CD107a and TNF-α. (**E**) Frequency of cells that express IFN-γ but not TNF-α or CD107a. (**F**) Frequency of cells that express CD107a but not IFN-γ or TNF-α. (**G**) Frequency of cells that express TNF-α but not IFN-γ or CD107a. (**H**) Frequency of cells that do not express IFN-γ, TNF-α or CD107a. *$P < 0.05$, **$P < 0.01$, ***$P < 0.001$, ****$P < 0.0001$ by paired two-way ANOVA and Tukey's multiple comparisons test. Each symbol represents a donor.

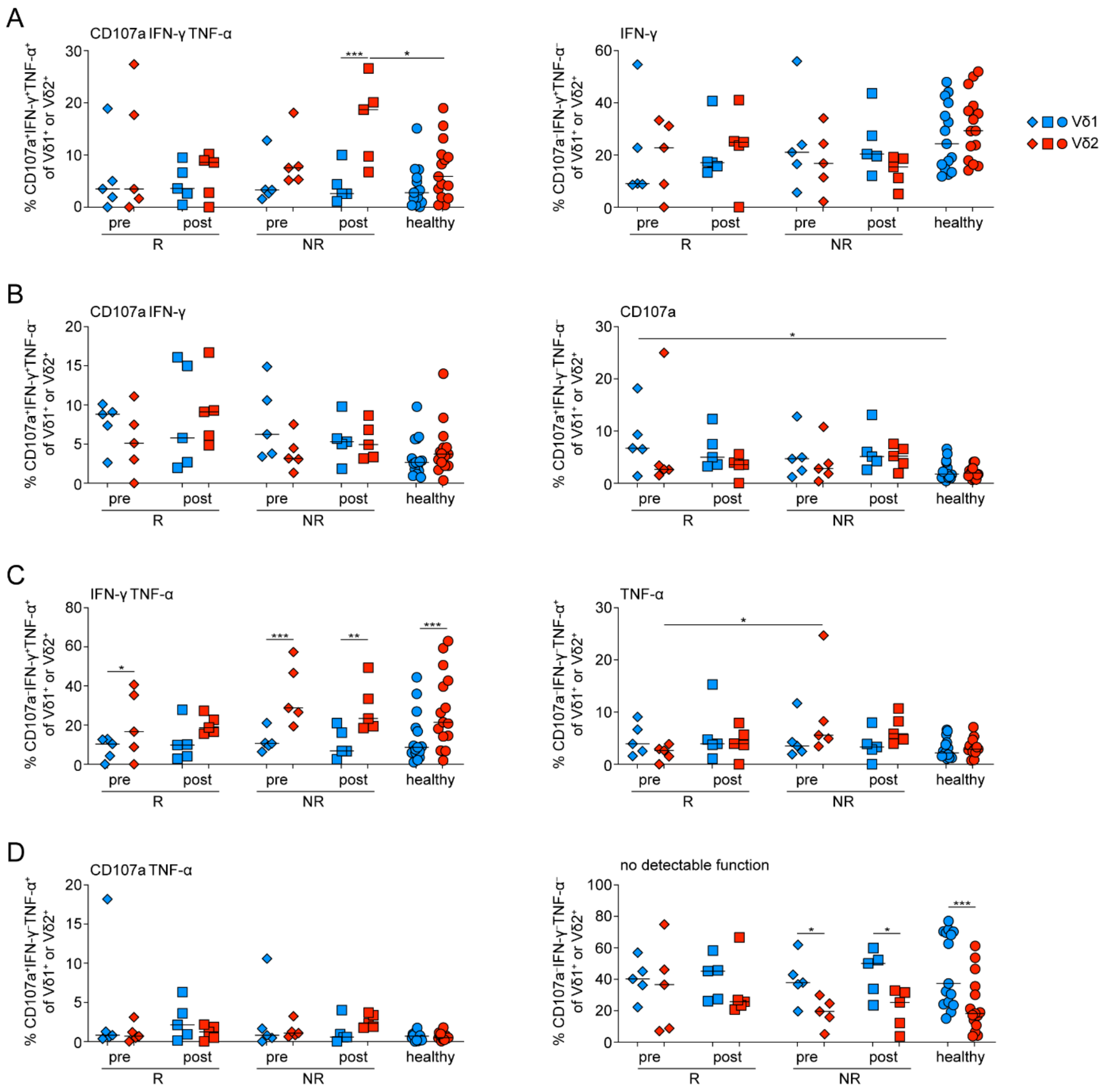

Appendix Figure S8. γδ T cell functionality after polyclonal activation in patients with melanoma treated with combination therapy. PBMCs from healthy donors (healthy) and patients with stage IV melanoma who responded (R) or did not respond (NR) to α-PD-1 and α-CTLA-4 combination treatment were stimulated for 3.5h to assess cytokine production. Patient samples from before (pre) and 3 to 4 months after (post) the start of treatment were analyzed. (**A**) Frequency of Vδ1 and Vδ2 cells that co-express CD107a, IFN-γ and TNF-α. (**B**) Frequency of cells that co-express CD107a and IFN-γ. (**C**) Frequency of cells that co-express IFN-γ and TNF-α. (**D**) Frequency of cells that co-express CD107a and TNF-α. (**E**) Frequency of cells that express IFN-γ but not TNF-α or CD107a. (**F**) Frequency of cells that express CD107a but not IFN-γ or TNF-α. (**G**) Frequency of cells that express TNF-α but not IFN-γ or CD107a. (**H**) Frequency of cells that do not express IFN-γ, TNF-α or CD107a. *P < 0.05, **P < 0.01, ***P < 0.001 by paired two-way ANOVA and Tukey's multiple comparisons test. Each symbol represents a donor.

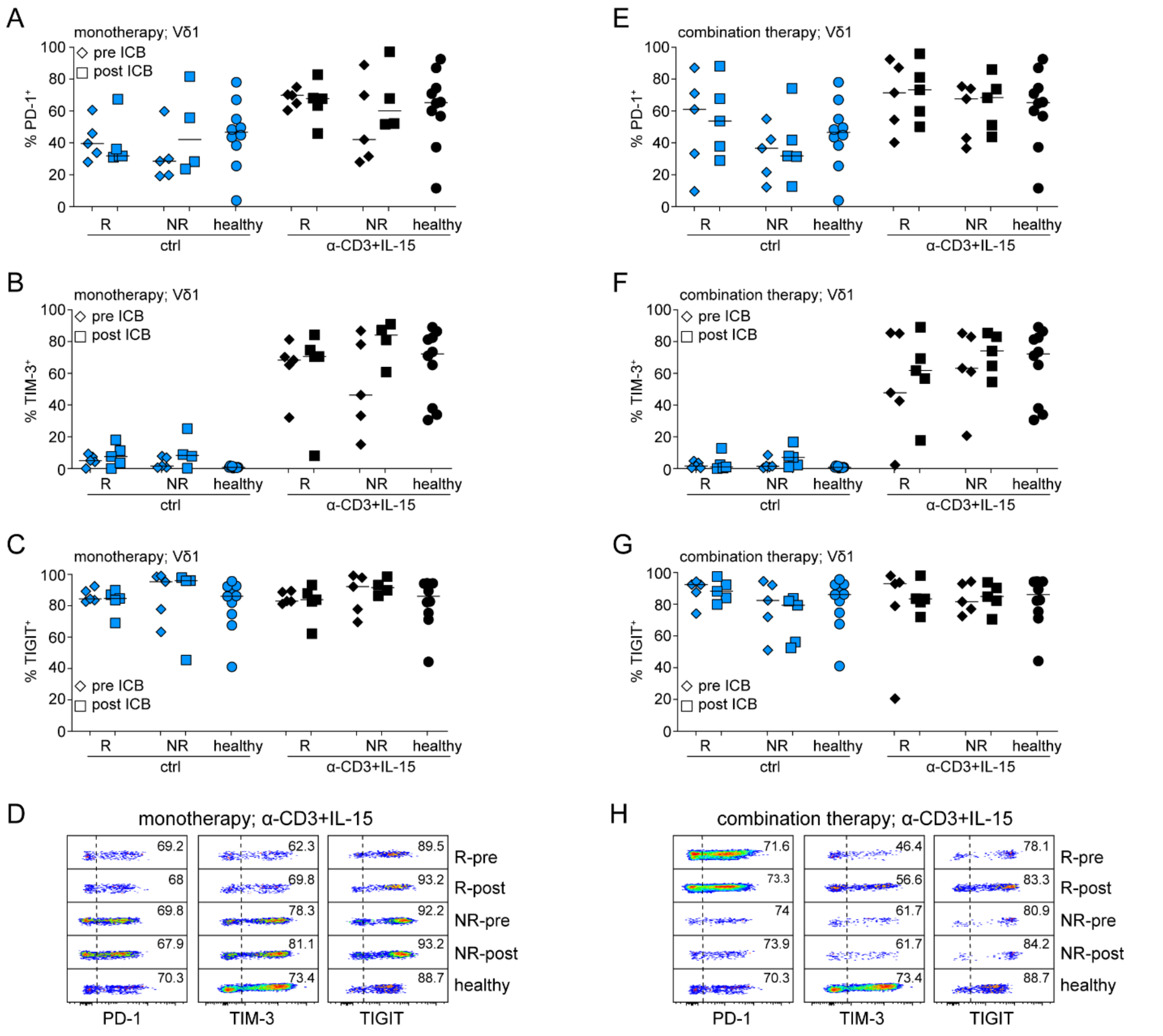

Appendix Figure S9. ICR expression in Vδ1 cells from patients with melanoma in response to TCR and IL-15 stimulation. Analysis of Vδ1 cells from patients with stage IV melanoma that were treated with either α-PD1 alone (monotherapy, **A-D**) or α-PD-1 and α-CTLA-4 (combination therapy; **E-H**). Samples were obtained from patients who responded (R) or did not respond (NR) to treatment both before (pre) and 3 to 4 months after (post) the start of immunotherapy. PBMCs were stimulated with α-CD3 and IL-15 for 48h before assessment of PD-1 (**A, E**), TIM-3 (**B, F**) and TIGIT (**C, G**) expression in Vδ1 cells. (**D, H**) Representative flow cytometric analysis in these samples. Statistical analysis by paired two-way ANOVA and Tukey's multiple comparisons test did not return statistical significance. In graphs, each symbol represents a donor.

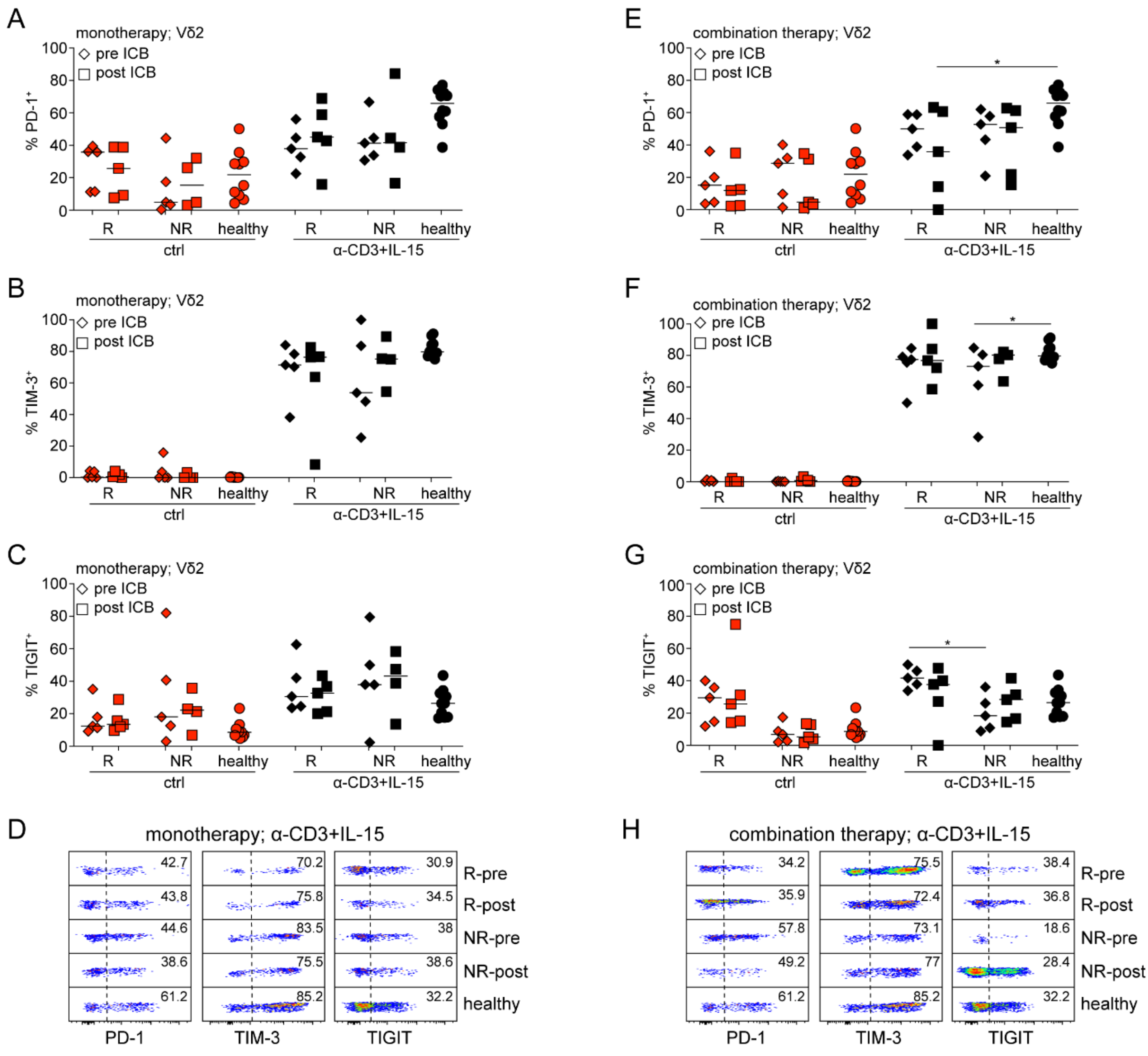

Appendix Figure S10. ICR expression in Vδ2 cells from patients with melanoma in response to TCR and IL-15 stimulation. Analysis of Vδ2 cells from patients with stage IV melanoma who were treated with either α-PD1 alone (monotherapy, **A-D**) or α-PD-1 and α-CTLA-4 (combination therapy; **E-H**). Samples were obtained from patients who responded (R) or did not respond (NR) to treatment both before (pre) and 3 to 4 months after (post) the start of immunotherapy. PBMCs were stimulated with α-CD3 and IL-15 for 48h before assessment of PD-1 (**A, E**), TIM-3 (**B, F**) and TIGIT (**C, G**) expression in Vδ1 cells. (**D, H**) Representative flow cytometric analysis in these samples. *P < 0.05, by paired two-way ANOVA and Tukey's multiple comparisons test. In graphs, each symbol represents a donor.

Appendix Table S1. Patient cohort in this study

		Cancer therapy		Clinical response	
	Total (N=28)	Pembrolizumab (N=18)	Nivolumab/Ipilimumab (N=10)	Responder (N=15)	Non-responder (N=13)
**Age (years)**					
Mean (SD)	66.6 (14.0)	72.8 (8.86)	55.4 (15.0)	68.5 (11.6)	64.5 (16.6)
Median [Min, Max]	71.5 [30.0, 84.0]	74.5 [51.0, 84.0]	55.0 [30.0, 73.0]	71.0 [43.0, 84.0]	72.0 [30.0, 83.0]
**Sex**					
Female	10 (35.7%)	6 (33.3%)	4 (40.0%)	7 (46.7%)	3 (23.1%)
Male	18 (64.3%)	12 (66.7%)	6 (60.0%)	8 (53.3%)	10 (76.9%)
**Melanoma type**					
Skin	25 (89.3%)	18 (100%)	7 (70.0%)	15 (100%)	10 (76.9%)
Ocular	2 (7.1%)	0 (0%)	2 (20.0%)	0 (0%)	2 (15.4%)
Unknown focus	1 (3.6%)	0 (0%)	1 (10.0%)	0 (0%)	1 (7.7%)
**Cancer stage**					
IV M1a	8 (28.6%)	6 (33.3%)	2 (20.0%)	5 (33.3%)	3 (23.1%)
IV M1b	4 (14.3%)	3 (16.7%)	1 (10.0%)	3 (20.0%)	1 (7.7%)
IV M1c	12 (42.9%)	9 (50.0%)	3 (30.0%)	4 (26.7%)	8 (61.5%)
IV M1d	4 (14.3%)	0 (0%)	4 (40.0%)	3 (20.0%)	1 (7.7%)
**Cancer therapy**					
Pembrolizumab or nivolumab	18 (64.3%)	18 (100%)	0 (0%)	10 (66.7%)	8 (61.5%)
Nivolumab/Ipilimumab	10 (35.7%)	0 (0%)	10 (100%)	5 (33.3%)	5 (38.5%)
**BRAF mut**					
No	13 (46.4%)	10 (55.6%)	3 (30.0%)	8 (53.3%)	5 (38.5%)
Yes	13 (46.4%)	8 (44.4%)	5 (50.0%)	7 (46.7%)	6 (46.2%)
n/a	2 (7.1%)	0 (0%)	2 (20.0%)	0 (0%)	2 (15.4%)
**Tumor PD-L1 status**					
Negative	16 (57.1%)	8 (44.4%)	8 (80.0%)	8 (53.3%)	8 (61.5%)
Positive (>1%)	9 (32.1%)	9 (50.0%)	0 (0%)	6 (40.0%)	3 (23.1%)
n/a	3 (10.7%)	1 (5.6%)	2 (20.0%)	1 (6.7%)	2 (15.4%)
**Best overall response**					
PD	12 (42.9%)	7 (38.9%)	5 (50.0%)	0 (0%)	12 (92.3%)
SD	2 (7.1%)	2 (11.1%)	0 (0%)	1 (6.7%)	1 (7.7%)
PR	5 (17.9%)	2 (11.1%)	3 (30.0%)	5 (33.3%)	0 (0%)
CR	9 (32.1%)	7 (38.9%)	2 (20.0%)	9 (60.0%)	0 (0%)
**Progression-free survival (days)**					
Mean (SD)	491 (428)	514 (449)	449 (408)	826 (305)	104 (41.4)
Median [Min, Max]	382 [77.0, 1230]	391 [77.0, 1230]	299 [77.0, 1090]	781 [306, 1230]	84.0 [77.0, 209]
**Overall survival (days)**					
Mean (SD)	689 (398)	763 (423)	556 (327)	920 (289)	422 (339)
Median [Min, Max]	647 [125, 1240]	933 [125, 1240]	511 [161, 1090]	1040 [476, 1240]	237 [125, 1090]
