# Supplementary material for: Dynamics of checkpoint receptors in γδ T cell subsets are associated with clinical response during anti-PD-1 immunotherapies
Source: EMBO Mol Med. 2025 Nov 27;18(1):91–119. doi: 10.1038/s44321-025-00338-9 (PMC12808275; doi:10.1038/s44321-025-00338-9)
Supplement: Supplementary file 9 — Expanded View Figures [file 44321_2025_338_MOESM9_ESM.pdf]

Expanded View Figures

Figure EV1. Differential regulation of ICRs in human γδ T cell subsets.

Flow cytometry analysis of PD-1, TIM-3, TIGIT, LAG-3, total (t)CTLA-4 and surface (s)CTLA-4 in Vδ1 (**A**) and Vδ2 (**B**) cells. PMBCs were stimulated for 48 h with α-CD3 or α-CD3 and IL-15 or left untreated (ctrl) in the presence of inhibitors as follows: abrocitinib was used for JAK1 inhibition; NSC33994 for JAK2 inhibition; JPX-0700 for STAT3/5 dual inhibition; and tofacitinib citrate was used at a concertation 10 nM to inhibit JAK3 signaling, and at a concentration of 1 µM for combined JAK1/2/3 inhibition. The heatmaps show average expression in cells from 13 different donors in four independent experiments. Detailed statistics for all possible comparisons in accompanying Appendix Fig. S2.

© The Author(s)

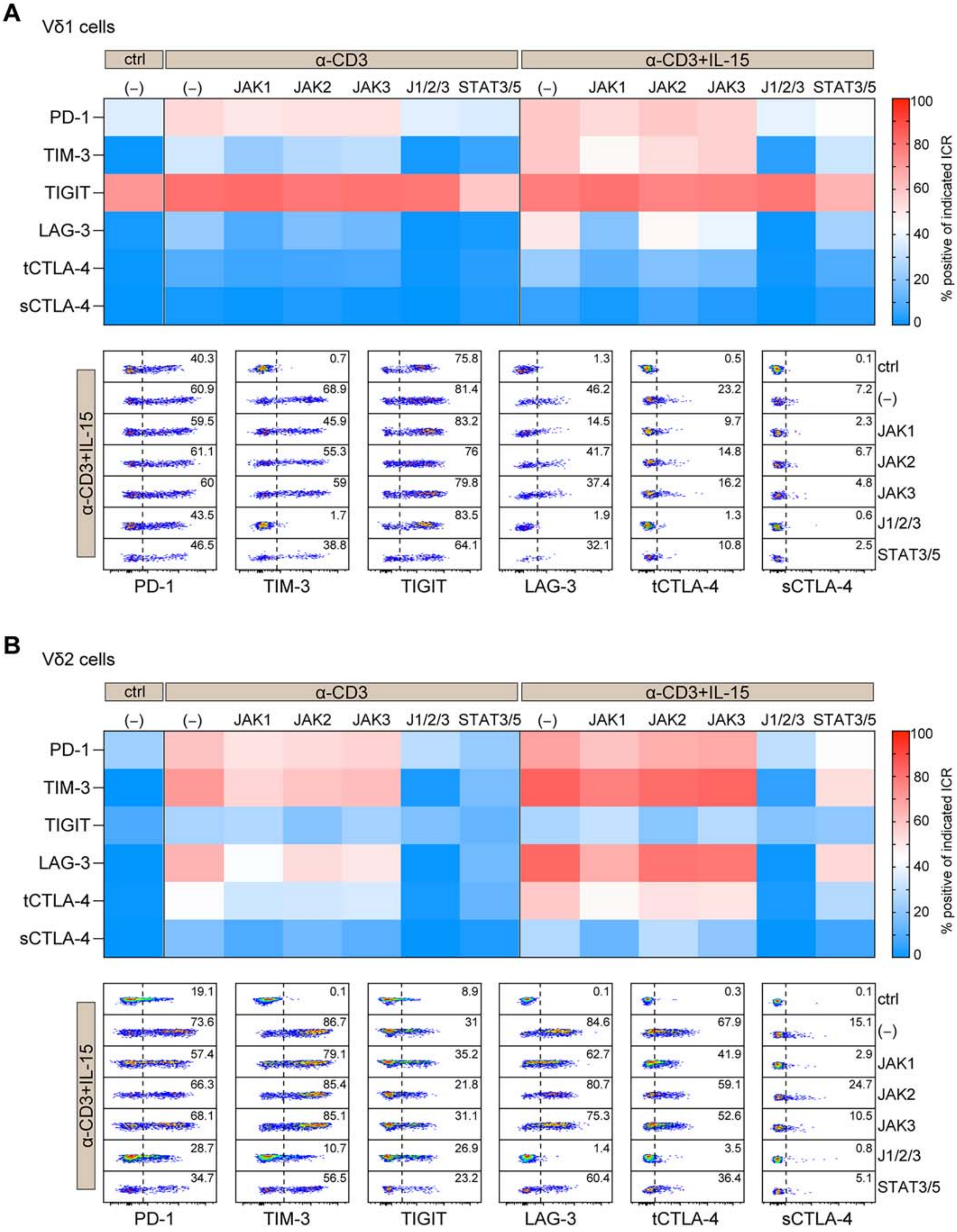
A
Vδ1 cells
ctrl
α-CD3
α-CD3+IL-15
(–) JAK1 JAK2 JAK3 J1/2/3 STAT3/5
PD-1
TIM-3
TIGIT
LAG-3
tCTLA-4
sCTLA-4
% positive of indicated ICR
0 20 40 60 80 100
α-CD3+IL-15
ctrl (–) JAK1 JAK2 JAK3 J1/2/3 STAT3/5
40.3 60.9 59.5 61.1 60 43.5 46.5
0.7 68.9 45.9 55.3 59 1.7 38.8
75.8 81.4 83.2 76 79.8 83.5 64.1
1.3 46.2 14.5 41.7 37.4 1.9 32.1
0.5 23.2 9.7 14.8 16.2 1.3 10.8
0.1 7.2 2.3 6.7 4.8 0.6 2.5
B
Vδ2 cells
19.1 73.6 57.4 66.3 68.1 28.7 34.7
0.1 86.7 79.1 85.4 85.1 10.7 56.5
8.9 31 35.2 21.8 31.1 26.9 23.2
0.1 84.6 62.7 80.7 75.3 1.4 60.4
0.3 67.9 41.9 59.1 52.6 3.5 36.4
0.1 15.1 2.9 24.7 10.5 0.8 5.1

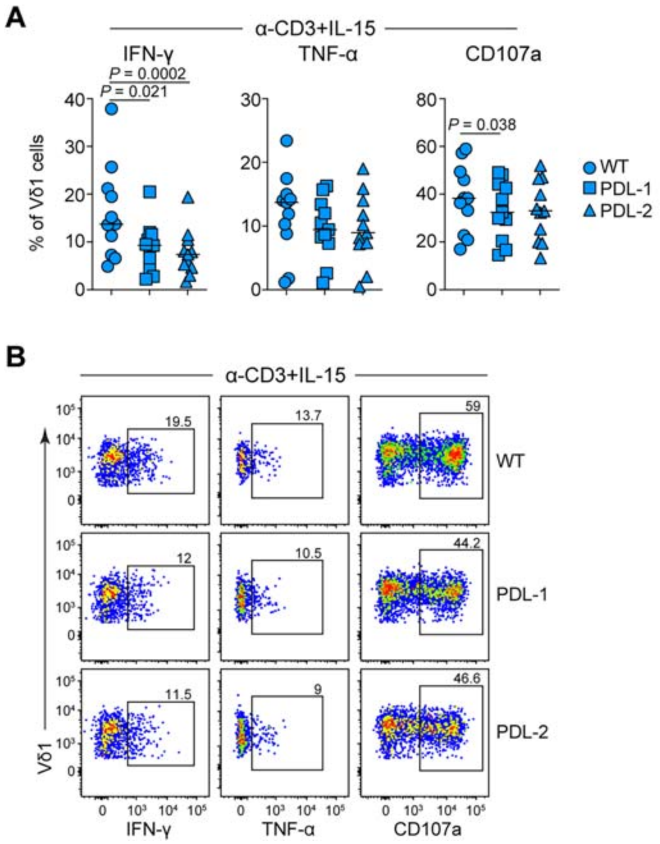

Figure EV2. PD-1 can inhibit the function of Vδ1 and Vδ2 cells.

PBMCs were magnetically depleted of $CD4^+$, $CD19^+$ and $CD14^+$ cells and cultured for 48 h with K562 cells expressing PDL-1, PDL-2 or no ligand (WT; wild-type), in the presence of IL-15 and α-CD3 stimulation. Levels of IFN-γ, TNF-α and CD107a were assessed by flow cytometry in Vδ1 (**A**, **B**) and Vδ2 (**C**, **D**) cells. Quantification (**A**, **C**) and representative plots (**B**, **D**) from 11 healthy donors in four independent experiments. *P* values were calculated by paired Friedman test with Dunn's multiple comparisons. In graphs, each symbol represents a donor.

 © The Author(s)

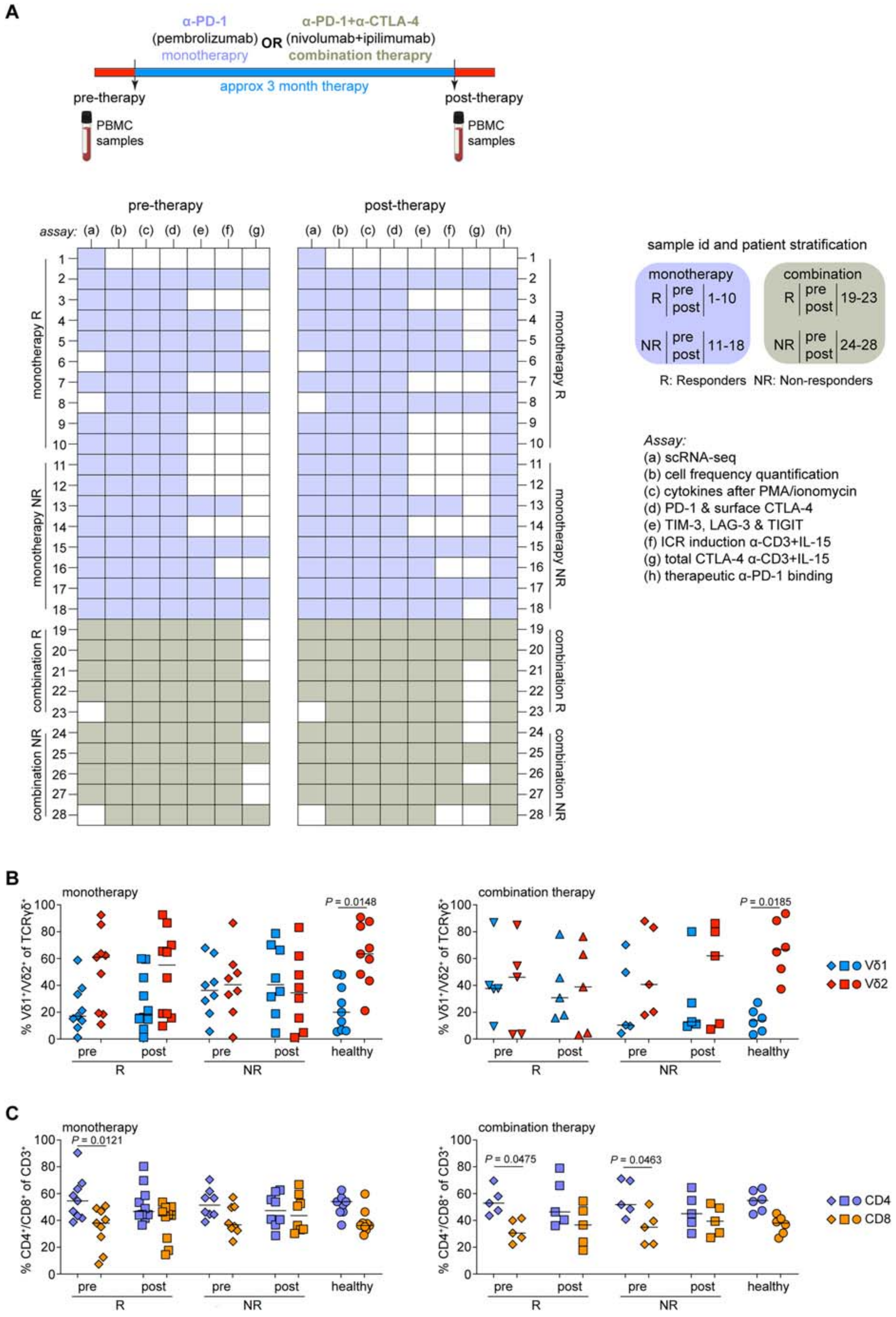
A
α-PD-1
(pembrolizumab)
monotherapy
OR
α-PD-1+α-CTLA-4
(nivolumab+ipilimumab)
combination therapy
approx 3 month therapy
pre-therapy
post-therapy
PBMC samples
sample id and patient stratification
monotherapy
R pre post 1-10
NR pre post 11-18
combination
R pre post 19-23
NR pre post 24-28
R: Responders NR: Non-responders
Assay:
(a) scRNA-seq
(b) cell frequency quantification
(c) cytokines after PMA/ionomycin
(d) PD-1 & surface CTLA-4
(e) TIM-3, LAG-3 & TIGIT
(f) ICR induction α-CD3+IL-15
(g) total CTLA-4 α-CD3+IL-15
(h) therapeutic α-PD-1 binding
monotherapy R
monotherapy NR
combination R
combination NR
B
monotherapy
combination therapy
% Vδ1+/Vδ2+ of TCRγδ+
P = 0.0148
P = 0.0185
pre post pre post healthy
R NR
Vδ1
Vδ2
C
% CD4+/CD8+ of CD3+
P = 0.0121
P = 0.0475
P = 0.0463
CD4
CD8

Figure EV3. ICB therapy study design and T cell frequencies in the various patient groups.

(**A**) PBMC samples from patients with stage IV melanoma who were treated with either α-PD1 alone (pembrolizumab; monotherapy) or α-PD-1 and α-CTLA-4 (nivolumab +ipilimumab; combination therapy) were analyzed as shown. Samples were taken pre- and post-therapy from patients who responded (R) or did not respond (NR) to treatment. (**B**, **C**) Flow cytometry analysis depicting frequencies of Vδ1 (blue) and Vδ2 (red) cells (**B**) or CD4 (purple) and CD8 (orange) T cells (**C**) derived from patients after monotherapy (left) or after combination therapy (right). *P* values were calculated by paired two-way ANOVA and Tukey's multiple comparisons test. In graphs, each symbol represents a donor.

 © The Author(s)

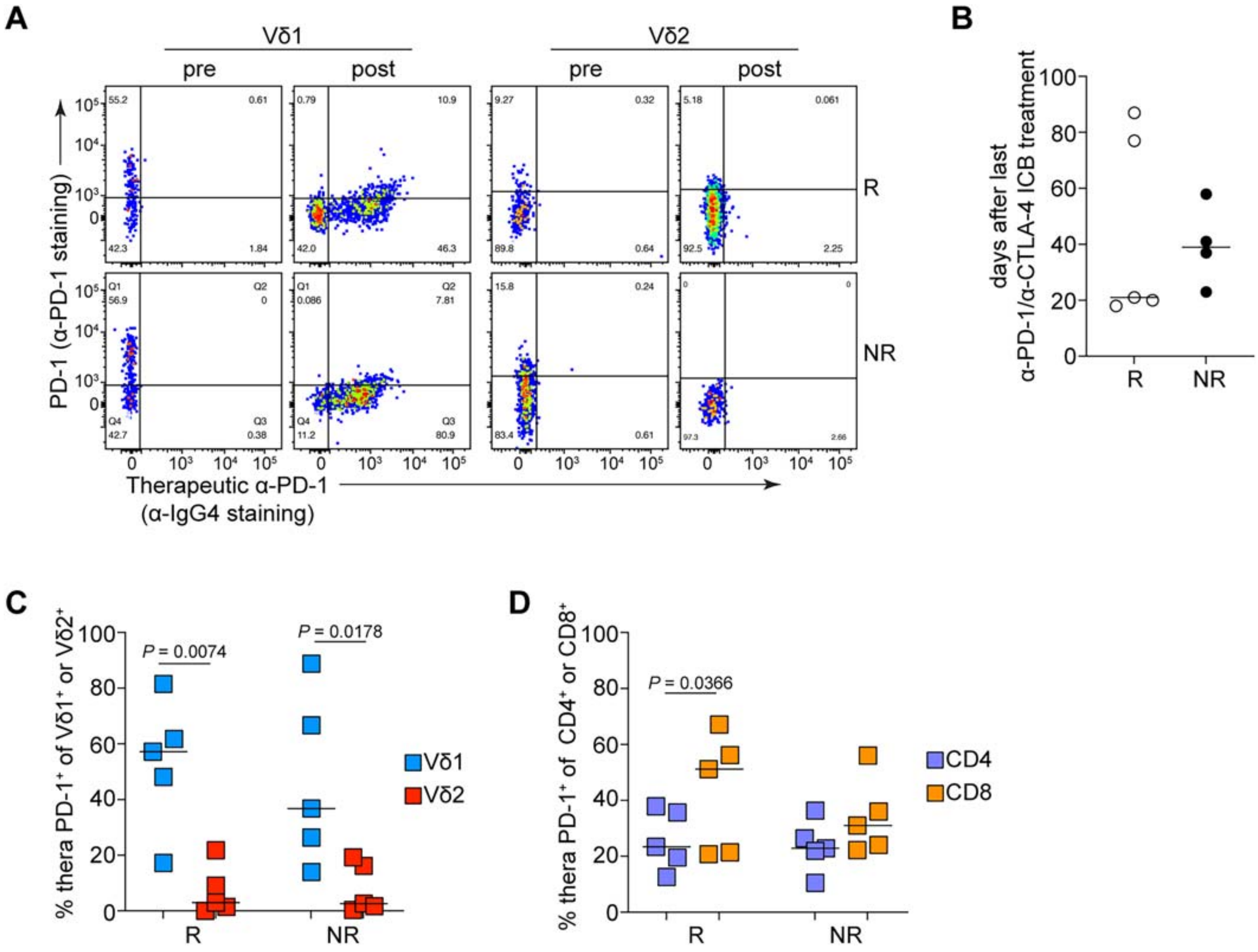

Figure EV4. Therapeutic antibody binding in patients undergoing α-PD1 and α-CTLA-4 combination therapy.

Analysis of PD-1 expression in PBMCs from patients with stage IV metastatic melanoma who either responded (R) or did not respond (NR) to a combination of nivolumab (therapeutic α-PD-1) and ipilimumab treatment. Paired samples were obtained from these patients before (pre) and 3 to 4 months after (post) the start of immunotherapy. The frequency of therapeutic antibody binding$^+$ (thera PD-1$^+$) cells was determined as explained in Fig. 2. (**A**) Representative flow cytometric analysis of therapeutic α-PD-1 antibody binding on the surface of Vδ1 and Vδ2 cells. (**B**) Days between the last antibody infusion and the collection of samples. (**C**) Thera PD-1$^+$ Vδ1 and Vδ2 cells after nivolumab+ipilimumab treatment. (**D**) Thera PD-1$^+$ CD4 and CD8 T cells after nivolumab+ipilimumab treatment. *P* values were calculated by paired two-way ANOVA and Tukey's multiple comparisons test. In graphs, each symbol represents a donor.

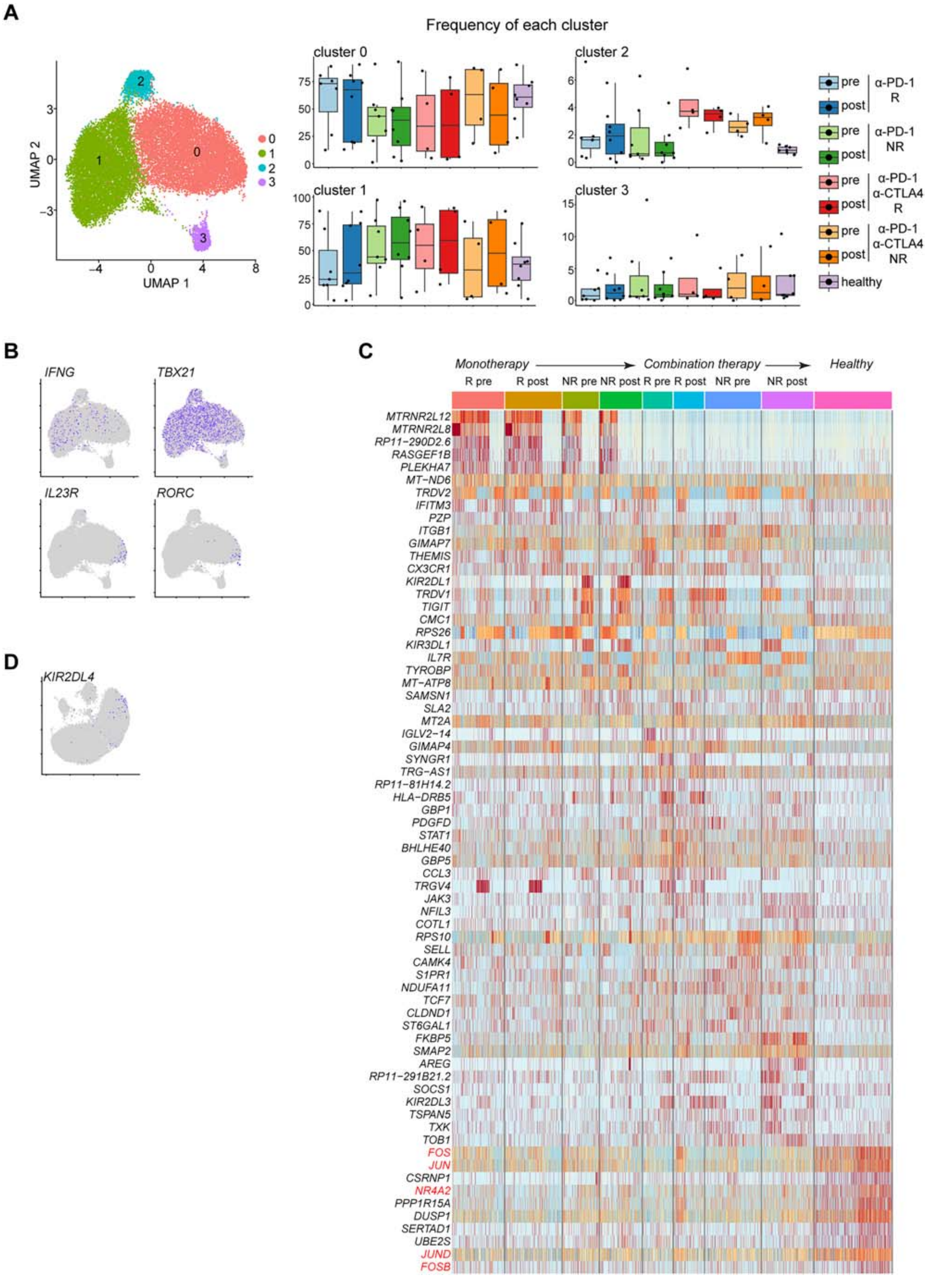
A
Frequency of each cluster
cluster 0
cluster 1
cluster 2
cluster 3
UMAP 1
UMAP 2
0
1
2
3
pre
post
α-PD-1 R
α-PD-1 NR
α-PD-1 α-CTLA4 R
α-PD-1 α-CTLA4 NR
healthy
B
IFNG
TBX21
IL23R
RORC
C
Monotherapy
Combination therapy
Healthy
R pre
R post
NR pre
NR post
R pre
R post
NR pre
NR post
MTRNR2L12
MTRNR2L8
RP11-290D2.6
RASGEF1B
PLEKHA7
MT-ND6
TRDV2
IFITM3
PZP
ITGB1
GIMAP7
THEMIS
CX3CR1
KIR2DL1
TRDV1
TIGIT
CMC1
RPS26
KIR3DL1
IL7R
TYROBP
MT-ATP8
SAMSN1
SLA2
MT2A
IGLV2-14
GIMAP4
SYNGR1
TRG-AS1
RP11-81H14.2
HLA-DRB5
GBP1
PDGFD
STAT1
BHLHE40
GBP5
CCL3
TRGV4
JAK3
NFIL3
COTL1
RPS10
SELL
CAMK4
S1PR1
NDUFA11
TCF7
CLDND1
ST6GAL1
FKBP5
SMAP2
AREG
RP11-291B21.2
SOCS1
KIR2DL3
TSPAN5
TXK
TOB1
FOS
JUN
CSRNP1
NR4A2
PPP1R15A
DUSP1
SERTAD1
UBE2S
JUND
FOSB
D
KIR2DL4

 © The Author(s)

Figure EV5. Transcriptomic analysis of circulating γδ T cells from patients with melanoma and healthy donors.

(**A**) UMAP embedding showing clustering of circulating γδ T cells ($n = 84{,}537$) derived from healthy donors ($n = 8$) and patients with stage IV melanoma who received α-PD-1 ($n = 16$) or a combination of α-PD-1 and α-CTLA-4 ($n = 8$) treatment (left), and the distribution of cells between clusters (right). To calculate the frequency of cells assigned to each cluster we only considered samples with at least 50 cells; each dot represents an individual sample. Each box represents the interquartile range showing 25th percentile, median and 75th percentile, and whiskers extend to the minimum and maximum values in the dataset. (**B**) Distribution of *IFNG, TBX21, IL23R*, and *RORC* expression in the dataset. (**C**) Heatmap showing normalized expression of the 10 most differentially expressed genes (DEGs) between patient groups. R, responder; NR, non-responder; pre, samples were taken before the start of treatment; post, samples were taken 3 to 4 months after the start of treatment. DEGs were identified by Wilcoxon Rank Sum test as those with an average Log2 fold change of at least 0.5 and an adjusted $P < 0.05$. (**D**) Distribution of *KIR2DL4* expression in the dataset.

© The Author(s)
